# Work productivity in real‐life employed patients with plaque psoriasis: Results from the ProLOGUE study

**DOI:** 10.1111/1346-8138.16517

**Published:** 2022-07-20

**Authors:** Hidehisa Saeki, Yasumasa Kanai, Kenta Murotani, Kei Ito, Takuya Miyagi, Hidetoshi Takahashi, Yayoi Tada, Mari Higashiyama, Yuki Hashimoto, Hiroki Kitabayashi, Shinichi Imafuku

**Affiliations:** ^1^ Department of Dermatology Nippon Medical School Tokyo Japan; ^2^ Medical Affairs Kyowa Kirin Co., Ltd. Tokyo Japan; ^3^ Biostatistics Center Kurume University Fukuoka Japan; ^4^ Department of Dermatology JR Sapporo Hospital Sapporo‐shi Japan; ^5^ Department of Dermatology University of the Ryukyus Okinawa Japan; ^6^ Takagi Dermatological Clinic Obihiro‐shi Japan; ^7^ Department of Dermatology Teikyo University School of Medicine Tokyo Japan; ^8^ Department of Dermatology Nippon Life Hospital Osaka Japan; ^9^ Department of Dermatology Toho University School of Medicine Tokyo Japan; ^10^ Department of Dermatology Fukuoka University Faculty of Medicine Fukuoka Japan

**Keywords:** brodalumab, employment, patient‐reported outcome measures, psoriasis, work performance

## Abstract

Psoriasis poses a substantial economic burden by reducing the work productivity of affected patients. We aimed to evaluate the negative impact of plaque psoriasis on work productivity and effectiveness of brodalumab in improving work productivity impairment in real‐life employed patients. This analysis was conducted in employed patients from ProLOGUE, an open‐label, multicenter, prospective cohort study (Japan Registry of Clinical Trials identifier: jRCTs031180037). Outcomes included association of Work Productivity and Activity Impairment‐Psoriasis (WPAI‐PSO) domain scores with scores from various patient‐reported outcome measures or Psoriasis Area and Severity Index (PASI) scores at baseline. Change from baseline in WPAI‐PSO domain scores following brodalumab treatment was also evaluated. Of the 73 patients enrolled, 51, 48, and 40 patients were considered employed at baseline, Week 12, and Week 48 of brodalumab treatment, respectively. In the model adjusted by age and sex, the work productivity loss score correlated with the Dermatology Life Quality Index (DLQI), itch Numeric Rating Scale (NRS), Patient Health Questionnaire‐8 (PHQ‐8), and skin pain NRS scores (partial Spearman correlation coefficient [*ρ*] = 0.608, 0.510, 0.461, and 0.424, respectively); presenteeism score correlated with the DLQI, itch NRS, and skin pain NRS scores (*ρ* = 0.568, 0.500, and 0.403, respectively); and activity impairment score correlated with the DLQI and PHQ‐8 scores (*ρ* = 0.530 and 0.414, respectively). None of the WPAI‐PSO domain scores correlated with the PASI score. All WPAI‐PSO domain scores (except absenteeism) significantly reduced from baseline to Weeks 12 (*p* < 0.0001) and 48 (*p* < 0.001) with brodalumab treatment. In conclusion, work productivity impairment in psoriasis was associated with various subjective symptoms that can be captured using patient‐reported outcome measures. Brodalumab treatment improved work productivity in real‐life employed patients with plaque psoriasis.

## 1 INTRODUCTION

Psoriasis is a chronic, immune‐mediated inflammatory skin disease affecting approximately 0.84% of the world's population (64.6 million individuals as of 2017)^1^ and 0.34% of the Japanese population, with 60.0% of the patients being men.^2^ Psoriasis not only has a profound negative impact on the physical, social, and psychological well‐being of patients^3^ but also poses a substantial economic burden on healthcare systems, patients, and their families.^4^, ^5^ A case–control study in Japan showed that most patients with psoriasis, especially men, present for their first clinical visit between 30 and 69 years of age,^6^ which are considered the most economically productive years of life. The costs of treating psoriasis with topical or systemic medication alone or biologics^7^, ^8^ and indirect costs associated with unemployment and reduced working ability add to the financial burden.^5^

Previous randomized controlled trials (RCTs) have demonstrated that biologic therapies are associated with significant improvement in work productivity/reduced work impairment and a reduction in associated costs in patients with moderate‐to‐severe psoriasis.^9^, ^10^, ^11^ However, there are limited reports on the effect of brodalumab, a human anti‐interleukin‐17 receptor monoclonal antibody approved for the treatment of plaque psoriasis,^12^, ^13^, ^14^ on work productivity.

Patient‐reported outcomes (PROs) are established tools used by physicians and patients for shared decision‐making on treatment preferences, optimizing treatment strategies and outcomes, and assessing patient satisfaction toward a treatment strategy.^15^, ^16^, ^17^ Work productivity outcomes are commonly assessed using the Work Productivity and Activity Impairment (WPAI) questionnaire—a PRO‐based quantitative measure of health‐related loss in work productivity.^18^, ^19^ It measures absenteeism (work time missed), presenteeism (impairment at work/reduced on‐the‐job effectiveness), work productivity loss (WPL; overall work impairment/absenteeism plus presenteeism), and activity impairment (AI; impairment in regular daily activities other than work) 7 days prior to questionnaire completion. WPAI has been adapted to specific diseases/health problems; WPAI‐Psoriasis (WPAI‐PSO) is a validated version of the questionnaire used to collect patient responses on the impact of psoriasis on work‐related and regular activities.^19^, ^20^

Therefore, using PROs to assess work productivity and effectiveness of biologic treatment in improving work productivity can provide further insights into the impact of psoriasis on real‐life patients. Using various PROs, the single‐arm, interventional, open‐label, multicenter, prospective ProLOGUE study^21^, ^22^ (Japan Registry of Clinical Trials identifier: jRCTs031180037) assessed the effectiveness of brodalumab in real‐life Japanese patients with plaque psoriasis. In this analysis of the ProLOGUE study, we assessed the associations between PROs and work productivity to evaluate the negative impact of plaque psoriasis on work productivity and the effectiveness of brodalumab in improving work productivity impairment in real‐life employed patients.

## 2 METHODS

### 2.1 Study design, patients, and treatment

The design and key eligibility criteria of the ProLOGUE study have been reported previously.^21^, ^22^ The ProLOGUE study was conducted at 15 facilities across Japan from October 2017 to March 2020 and included patients (aged ≥18 years) with plaque psoriasis who had no peripheral arthritis symptoms, could self‐administer brodalumab, and had not appropriately responded to existing systemic treatments.^21^ Patients received brodalumab 210 mg subcutaneously on Day 1 and at Weeks 1 and 2, followed by once every 2 weeks. Patients were treated in daily clinical practice per the Japanese drug package insert, and no criteria were set for concomitant or prohibited therapies.^22^

This study was first reviewed and approved by the research ethics committee of each participating facility. Following enforcement of the Japanese act for the conduct of clinical research funded by pharmaceutical companies in April 2018, the study was reviewed and approved by the Certified Review Board of Nippon Medical School Foundation. All patients provided written informed consent for participation in the study.

### 2.2 Outcomes

The outcomes assessed were correlations between WPAI‐PSO domain scores and scores from other PRO measures or the Psoriasis Area and Severity Index (PASI) scores at baseline, change from baseline in WPAI‐PSO domain scores following brodalumab treatment, and income opportunity loss because of WPL in employed patients.

### 2.3 Assessments

Patients' employment status and work‐associated problems due to psoriasis during the preceding 7 days were assessed at baseline and at Weeks 12 and 48 of brodalumab treatment using the WPAI‐PSO questionnaire^19^ with the help of an electronic PRO system. The WPAI‐PSO questionnaire comprised six questions. Patients who responded “yes” to the WPAI‐PSO question 1 “Are you currently employed (working for pay)?” were deemed employed. Patients answering “yes” to question 1 at baseline; at baseline and Week 12; and at baseline, Week 12, and Week 48 were considered employed at baseline, Week 12, and Week 48, respectively. The domain scores for absenteeism, presenteeism, WPL, and AI were calculated on a scale of 0.0%–100.0% each, with higher scores indicating more impairment or lower productivity.^19^ Patients reporting a score of greater than 0.0% in any domain were considered as having impairment in that WPAI‐PSO domain.

Other scores that were captured using an electronic PRO system before baseline medical examination were current dermatology‐specific health‐related quality of life (HRQoL), measured using the Dermatology Life Quality Index (DLQI);^23^ itching and skin pain levels during the preceding 24 h, assessed using the itch Numeric Rating Scale (NRS)^24^, ^25^ and skin pain NRS^26^ scores, respectively (range: 0 [no itch/pain] to 10 [worst imaginable itch/pain]); patients' satisfaction with their medication over the preceding 2–3 weeks or since the most recent use, assessed using the Treatment Satisfaction Questionnaire for Medication‐9 (TSQM‐9) domain scores (effectiveness, convenience, and global satisfaction; 0.0%–100.0% each);^27^ anxiety and depressive symptoms during the preceding 2 weeks, assessed using the Generalized Anxiety Disorder‐7 (GAD‐7) score (range, 0–21)^28^ and the Patient Health Questionnaire‐8 (PHQ‐8) score (range, 0–24),^29^ respectively (higher scores indicating more severe symptoms); sleep problems during the preceding 4 weeks, assessed using the Sleep Problems Index‐II (SPI‐II) score (range, 0–100; higher scores indicating fewer sleep‐related problems);^30^ and general health status, assessed on the day of the survey using the European Quality of Life 5‐Dimensions 5‐Levels Utility Index (EQ‐5D‐5L UI) score (range, −0.025 to 1.000; higher scores indicating higher health utility), calculated using the Japanese tariff.^31^ The PASI score was assessed by the attending physician.

### 2.4 Statistical analyses

All patients except those who did not receive brodalumab were included in the full analysis set. This analysis was conducted in those patients in the full analysis set who were employed according to question 1 of the WPAI‐PSO questionnaire. Correlations between each WPAI‐PSO domain score and the PASI score as well as scores from other PRO measures were assessed using the partial Spearman correlation coefficient ([*ρ*]; absolute value ≥0.70, strongly correlated; ≥0.40 to <0.70, correlated; ≥0.20 to <0.40, weakly correlated; <0.20, not correlated),^32^ with a significance level of *p* < 0.05 (two‐tailed). The covariates used for adjustment were age and sex. The WPAI‐PSO domain scores and PASI and DLQI scores of employed patients at baseline were compared with those at Weeks 12 and 48 of brodalumab treatment using the Wilcoxon signed‐rank test.

Based on the 2017 national wage data in Japan published by the National Tax Agency, Commissioner's Secretariat, Planning Division (stratified by age category and sex),^33^ the income opportunity loss (loss of annual income per person owing to WPL) at baseline and Week 48 for each employed patient was calculated using the reference value that matched the age category (20–24, 25–29, 30–34, 35–39, 40–44, 45–49, 50–54, 55–59, 60–64, 65–69, and ≥70 years) or sex.

Analyses were performed using last observation carried forward, where discontinuations (patients who withdrew from the study) up to Week 12 were recorded as Week 12 data and those between Weeks 12 and 48 were recorded as Week 48 data. No other imputation for missing values was performed. In addition, all analyses were exploratory in nature; hence, the analysis did not account for multiple comparisons. All analyses were performed using SAS version 9.4 (SAS Institute, Inc., Cary, NC, USA).

## 3 RESULTS

### 3.1 Patient disposition and baseline characteristics

**FIGURE 1:**
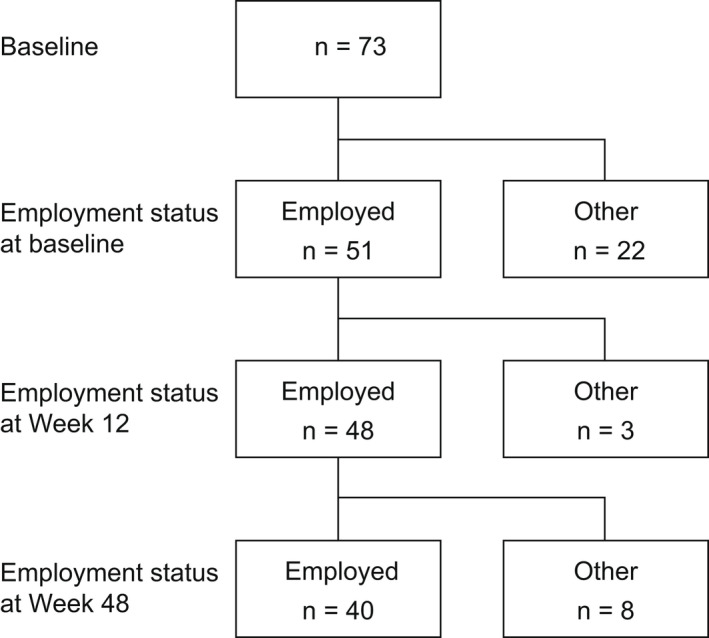
Patient disposition. “Employed” refers to patients who responded “yes” to question 1 of the WPAI‐PSO questionnaire. “Other” refers to patients who responded “no” to question 1 of the WPAI‐PSO questionnaire.^19^ WPAI‐PSO, Work Productivity and Activity Impairment‐Psoriasis.

Of the 73 patients originally enrolled in this study,^21^ 51 (69.9%), 48 (65.8%), and 40 (54.8%) were deemed employed at baseline, Week 12, and Week 48, respectively (Figure 1). Among the employed patients at baseline, 44 (86.3%) were men. The median (quartile [Q]1–Q3) age was 54.0 (42.0–62.0) years; body weight, 70.0 (63.0–80.2) kg; and body mass index, 24.5 (22.9–28.0) kg/m^2^ (Table 1). Median (Q1–Q3) PASI, DLQI, and WPAI‐PSO AI scores at baseline were 12.6 (9.2–15.3), 7.0 (3.0–10.0), and 10.0% (0.0%–50.0%), respectively.

**TABLE 1:** Baseline demographics and characteristics of employed patients

Category	All patients (*n* = 51)
Male	44 (86.3)
BMI^a^, ≥25 kg/m^2^	22 (45.8)
Smoking	37 (72.5)
Drinking	46 (90.2)
Prior biologics use	21 (41.2)
Age, years	54.0 (42.0–62.0)
Disease duration, years	16.0 (8.0–24.0)
Age of onset, years	33.0 (25.0–48.0)
Body weight^a^, kg	70.0 (63.0–80.2)
BMI^a^, kg/m^2^	24.5 (22.9–28.0)
PASI score	12.6 (9.2–15.3)
DLQI score	7.0 (3.0–10.0)
WPAI‐PSO AI, %	10.0 (0.0–50.0)

*Note:* Data are presented as *n* (%) or median (Q1–Q3).

Abbreviations: BMI, body mass index; DLQI, Dermatology Life Quality Index; PASI, Psoriasis Area and Severity Index; Q, quartile; WPAI‐PSO AI, activity impairment domain of the Work Productivity and Activity Impairment‐Psoriasis questionnaire.

^a^ *n* = 48 as body weight and BMI data were missing for three patients.

### 3.2 Correlation between WPAI‐PSO domain scores and PRO and PASI scores at baseline

In the adjusted model, the WPL score correlated with the DLQI, itch NRS, PHQ‐8, and skin pain NRS scores (*ρ* = 0.608, 0.510, 0.461, and 0.424, respectively) and weakly correlated with the GAD‐7, SPI‐II, EQ‐5D‐5L UI, TSQM‐9 effectiveness, and TSQM‐9 convenience scores (*ρ* = 0.329, −0.372, −0.330, −0.309, and − 0.283, respectively; Table 2). No correlation was found between the WPL and TSQM‐9 global satisfaction scores (*ρ* = −0.189). The presenteeism score correlated with the DLQI, itch NRS, and skin pain NRS scores (*ρ* = 0.568, 0.500, and 0.403, respectively) and weakly correlated with the PHQ‐8, GAD‐7, TSQM‐9 convenience, EQ‐5D‐5L UI, and SPI‐II scores (*ρ* = 0.358, 0.309, −0.286, −0.285, and − 0.283, respectively). In contrast, the absenteeism score weakly correlated with only the PHQ‐8 and SPI‐II scores (*ρ* = 0.367 and − 0.390, respectively). The AI score correlated with the DLQI and PHQ‐8 scores (*ρ* = 0.530 and 0.414, respectively) and weakly correlated with the GAD‐7 and TSQM‐9 convenience scores (*ρ* = 0.337 and − 0.298, respectively).

**TABLE 2:** Correlation (*ρ*) between WPAI‐PSO domain scores and symptom‐related, HRQoL‐related, and satisfaction‐related PRO and PASI scores at baseline

Scores	Unadjusted model				Adjusted model			
	WPL	Presenteeism	Absenteeism	AI	WPL	Presenteeism	Absenteeism	AI
PASI	0.335 (0.0161)	0.36 (0.0094)	0.242 (0.0872)	0.363 (0.0088)	0.228 (0.1155)	0.253 (0.0789)	0.244 (0.0905)	0.251 (0.0814)
Itch NRS	0.527 (<0.0001)	0.516 (0.0001)	0.133 (0.3516)	0.329 (0.0184)	0.51 (0.0002)	0.5 (0.0003)	0.127 (0.3831)	0.277 (0.0536)
Skin pain NRS	0.443 (0.0011)	0.423 (0.002)	0.153 (0.2833)	0.263 (0.0626)	0.424 (0.0024)	0.403 (0.0041)	0.145 (0.3208)	0.209 (0.149)
GAD‐7	0.429 (0.0017)	0.415 (0.0025)	0.137 (0.3394)	0.445 (0.0011)	0.329 (0.0209)	0.309 (0.0307)	0.128 (0.3809)	0.337 (0.0178)
PHQ‐8	0.523 (<0.0001)	0.441 (0.0012)	0.352 (0.0114)	0.481 (0.0004)	0.461 (0.0009)	0.358 (0.0115)	0.367 (0.0095)	0.414 (0.0031)
SPI‐II	−0.333 (0.017)	−0.253 (0.0736)	−0.388 (0.0049)	−0.153 (0.2847)	−0.372 (0.0084)	−0.283 (0.0485)	−0.39 (0.0055)	−0.174 (0.2311)
DLQI	0.692 (<0.0001)	0.667 (<0.0001)	0.203 (0.1534)	0.641 (<0.0001)	0.608 (<0.0001)	0.568 (<0.0001)	0.228 (0.1143)	0.53 (<0.0001)
EQ‐5D‐5L UI	−0.364 (0.0087)	−0.325 (0.02)	−0.146 (0.305)	−0.287 (0.0409)	−0.33 (0.0204)	−0.285 (0.0474)	−0.147 (0.3149)	−0.244 (0.0913)
TSQM‐9: effectiveness	−0.267 (0.0585)	−0.236 (0.0961)	−0.176 (0.218)	−0.162 (0.2553)	−0.309 (0.0308)	−0.275 (0.0557)	−0.189 (0.1943)	−0.206 (0.1552)
TSQM‐9: convenience	−0.349 (0.012)	−0.354 (0.0108)	−0.067 (0.6391)	−0.357 (0.0102)	−0.283 (0.0487)	−0.286 (0.0463)	−0.074 (0.6133)	−0.298 (0.0373)
TSQM‐9: global satisfaction	−0.108 (0.4514)	−0.077 (0.5914)	−0.078 (0.5852)	0.037 (0.7949)	−0.189 (0.1934)	−0.157 (0.2803)	−0.09 (0.5395)	−0.036 (0.8046)

*Note:* Data are presented as *ρ* (*p* value).

Abbreviations: AI, activity impairment; DLQI, Dermatology Life Quality Index; EQ‐5D‐5L UI, European Quality of Life 5‐Dimensions 5‐Levels Utility Index; GAD‐7, Generalized Anxiety Disorder‐7; HRQoL, health‐related quality of life; NRS, Numeric Rating Scale; PASI, Psoriasis Area and Severity Index; PHQ‐8, Patient Health Questionnaire‐8; PRO, patient‐reported outcome; SPI‐II, Sleep Problems Index‐II; TSQM‐9, Treatment Satisfaction Questionnaire for Medication‐9; WPAI‐PSO, Work Productivity and Activity Impairment‐Psoriasis; WPL, work productivity loss; *ρ*, partial Spearman correlation coefficient.

None of the WPAI‐PSO domain scores showed correlation with the PASI score in the adjusted analysis, although a weak correlation was observed between the PASI and WPL, presenteeism, and AI domain scores in the unadjusted analysis.

### 3.3 Work productivity impairment

**FIGURE 2:**
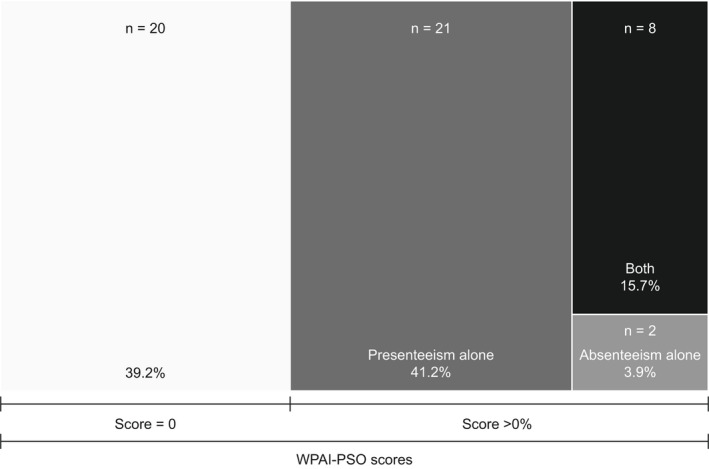
Work productivity impairment in employed patients at baseline. Data represent patients with work productivity impairment (WPAI‐PSO score >0.0%) in the absenteeism or presenteeism domains. WPAI‐PSO, Work Productivity and Activity Impairment‐Psoriasis.

Of the 51 employed patients at baseline, 31 (60.8%) reported WPL (score >0.0% either in the WPAI‐PSO absenteeism and/or presenteeism domains), with 21 (41.2%), 2 (3.9%), and 8 (15.7%) patients reporting impairment because of presenteeism alone, absenteeism alone, and both absenteeism and presenteeism, respectively (Figure 2). Impairment in the WPAI‐PSO AI domain (score >0.0%) was reported by 32 (62.7%) patients (data not shown).

### 3.4 Change from baseline in WPAI‐PSO domain scores following brodalumab treatment

**FIGURE 3:**
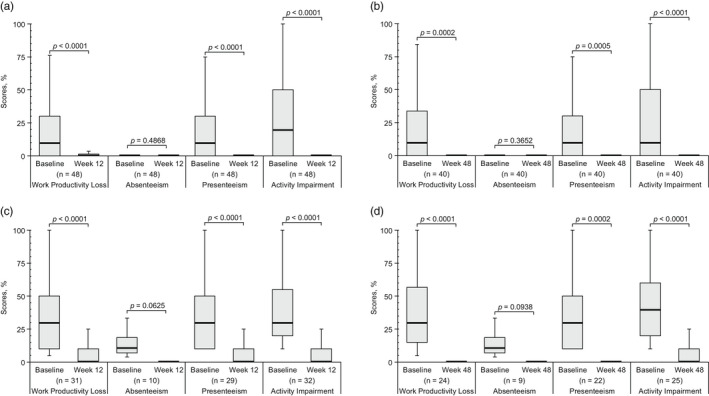
Change from baseline in the WPAI‐PSO domain scores: (a) baseline vs Week 12 in all employed patients, (b) baseline vs Week 48 in all employed patients, (c) baseline vs Week 12 in employed patients with impaired WPAI‐PSO domain score (>0.0%) at baseline, and (d) baseline vs Week 48 in employed patients with impaired WPAI‐PSO domain score (>0.0%) at baseline. Top whisker represents Q3 + (1.5 × IQR) or the maximum, whichever is lower; bottom whisker represents Q1 − (1.5 × IQR) or the minimum, whichever is higher; top border of the box represents Q3; bottom border of the box represents Q1; middle (bolded) line of the box represents the median. IQR, interquartile range; Q, quartile; WPAI‐PSO, Work Productivity and Activity Impairment‐Psoriasis.

A statistically significant reduction from baseline in all WPAI‐PSO domain scores, except absenteeism (*p* = 0.4868), was observed at Week 12 (*p* < 0.0001 for WPL, presenteeism, and AI, each; Figure 3a) and Week 48 (*p* = 0.0002, 0.0005, and <0.0001 for WPL, presenteeism, and AI, respectively; Figure 3b) of brodalumab treatment. In employed patients with impaired WPAI‐PSO domain scores (>0.0%) at baseline, WPL, presenteeism, and AI scores significantly decreased from baseline to Week 12 (*p* < 0.0001 for all; Figure 3c) and Week 48 (WPL, *p* < 0.0001; presenteeism, *p* = 0.0002; AI, *p* < 0.0001; Figure 3d) of brodalumab treatment. The absenteeism score did not show any significant reduction in employed patients, irrespective of their WPAI‐PSO scores at baseline. In employed patients with no impairment in the WPAI‐PSO domain scores (0.0%) at baseline, no significant difference from baseline was observed following brodalumab treatment (data not shown). The PASI and DLQI scores of employed patients significantly decreased from baseline to Weeks 12 and 48 (*p* < 0.0001 for each) of brodalumab treatment (Table S1 in Appendix S1).

### 3.5 Income opportunity loss

**FIGURE 4:**
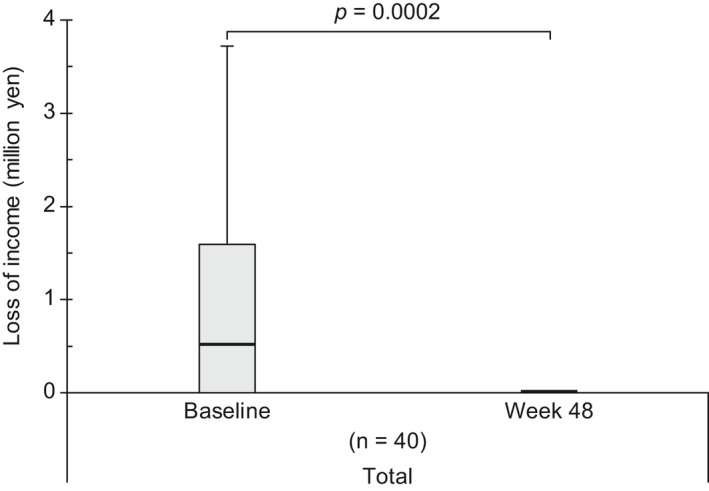
Change in income opportunity loss (loss of annual income per person) because of WPL from baseline to Week 48 in all employed patients. Top whisker represents Q3 + (1.5 × IQR) or the maximum, whichever is lower; bottom whisker represents Q1 – (1.5 × IQR) or the minimum, whichever is higher; top border of the box represents Q3; bottom border of the box represents Q1; middle (bolded) line of the box represents the median. IQR, interquartile range; Q, quartile; WPL, work productivity loss.

Income opportunity loss owing to WPL in employed patients significantly decreased from baseline to Week 48 (*p* = 0.0002) of brodalumab treatment (Figure 4). Although income opportunity loss was observed to numerically decrease at Week 48 of brodalumab treatment on stratifying patients by age (25–44 and 45–64 years) and sex (Figure S1), statistical analyses to confirm these findings were not performed. Furthermore, no income opportunity loss owing to WPL at both baseline and Week 48 was observed in patients aged ≥65 years (Figure S1) because the WPL domain score was 0.0% in this age group (data not shown).

## 4 DISCUSSION

Work productivity is a significant challenge for employed patients with psoriasis.^34^, ^35^, ^36^ Treatment with biologics has shown efficacy in improving work productivity in patients with moderate‐to‐severe psoriasis in several RCTs.^10^, ^37^, ^38^ Results of the current analysis of the ProLOGUE study confirmed improved work productivity in Japanese employed patients with psoriasis who were treated with brodalumab in daily clinical practice, as illustrated by reductions in the WPL, presenteeism, and AI domain scores from baseline to Weeks 12 and 48 of brodalumab treatment.

In the current analysis, approximately 40% of the employed patients at baseline did not show WPL. The median baseline WPL score was 10.0% for all employed patients and 30.0% for employed patients with impaired WPAI‐PSO domain score at baseline, which is lower than the mean WPL score at baseline (40.2%) reported in a post‐marketing surveillance of a biologic agent in Japanese patients with psoriatic arthritis.^39^ The difference in the baseline WPL score between the studies can be attributed to the exclusion of patients with peripheral arthritis from the present study because previous research has suggested an association between psoriatic arthritis and reduced work productivity.^40^, ^41^ In contrast to previous research findings derived from patients with psoriasis treated with biologics,^41^, ^42^ no association was observed between improvement in the PASI score and reduction in WPL in the current analysis, which could be supported by results of other real‐life studies.^39^, ^43^ The lack of association between these two factors observed in some of the real‐life studies, including ours, can be attributed to lower baseline PASI scores in real‐life settings (mean, 9.0–9.5;^39^, ^43^ median, 12.1^21^) compared with those recorded in the RCT (mean, 21.5–21.7)^44^ or the use of models adjusted by age and sex in our analysis and a previous real‐life study.^43^ Indeed, in the unadjusted model of the current analysis, the PASI score and WPL domain score showed a weak correlation.

We observed a decrease in income opportunity loss due to WPL in employed patients with psoriasis following brodalumab treatment, which remained consistent in patients aged <65 years. As psoriasis is more common in patients aged 30–69 years,^6^ an aggressive therapy including treatment with biologics may be needed for patients in their prime working years to improve their clinical condition and minimize indirect costs resulting from WPL.

Our study has some limitations. First, the number of patients, especially women, with an impaired absenteeism domain score at baseline was limited. Second, we did not collect information on patients' working status (part‐ or full‐time) or type of occupation, making it difficult to evaluate the impact of these factors on the current results. Lastly, our results are indicative of only a partial cost to patients with psoriasis because they only show the inferred opportunity loss by the patients themselves (the expected impact on their income), not accounting for other expenses such as indirect (caregivers and transportation) and direct (medical fees) costs.

In summary, the impairment in work productivity in real‐life employed patients with psoriasis was associated with various subjective symptoms that can be captured using PROs. Therefore, WPAI‐PSO can be an important source of information for shared decision‐making between patients with psoriasis, most of whom are in their prime working years, and physicians for choosing and optimizing treatment options. Treatment with brodalumab resulted in improved work productivity and minimized annual income loss due to WPL in employed patients with psoriasis.

## CONFLICT OF INTEREST

H. Saeki reports grants from Kyowa Kirin during the study period; grants, personal fees, and nonfinancial support from Kyowa Kirin, Mitsubishi Tanabe Pharma, Taiho Pharmaceutical, Maruho, TOKIWA Pharmaceutical, Torii Pharmaceutical, and Eisai outside the submitted work; and personal fees and nonfinancial support from Sanofi, Celgene, and KYORIN Pharmaceutical outside the submitted work. Y. Kanai is an employee of Kyowa Kirin. K. Murotani reports grants from Kyowa Kirin during the study period. K. Ito reports grants from Kyowa Kirin during the study period and personal fees from Kyowa Kirin, Mitsubishi Tanabe Pharma, Sato Pharmaceutical, Ushio, Amgen, Janssen Pharmaceutical, AbbVie, Eisai, Sanofi, Eli Lilly Japan, Maruho, Nippon Kayaku, Taiho Pharmaceutical, and Novartis Pharma outside the submitted work. T. Miyagi reports grants from Kyowa Kirin during the study period; grants, personal fees, and nonfinancial support from AbbVie, Kaken Pharmaceutical, Maruho, Boehringer Ingelheim, Eisai, Celgene, Eli Lilly Japan, Novartis Pharma, and Taiho Pharmaceutical outside the submitted work; grants and personal fees from Daiichi Sankyo, Sanofi, Ono Pharmaceutical, Mitsubishi Tanabe Pharma, and Otsuka Pharmaceutical outside the submitted work; personal fees and nonfinancial support from Janssen Pharmaceutical; grants from Actelion, Earth Corporation, Teijin Pharma, LEO Pharma, and Sato Pharmaceutical outside the submitted work; and personal fees from CSL Behring outside the submitted work. H. Takahashi reports grants from Kyowa Kirin during the study period. Y. Tada reports grants from Kyowa Kirin during the study period; grants, personal fees, and nonfinancial support from Kyowa Kirin, Eli Lilly Japan, AbbVie, Maruho, Celgene, Taiho Pharmaceutical, Mitsubishi Tanabe Pharma, Novartis Pharma, Sanofi, UCB Japan, Torii Pharmaceutical, LEO Pharma, Eisai, Kaken Pharmaceutical, Pfizer, Ushio, Meiji Seika Pharma, Nippon Boehringer Ingelheim, JIMRO, Bristol Myers Squibb, and TOKIWA Pharmaceutical outside the submitted work; grants and nonfinancial support from Kanebo Cosmetics, MSD, Ono Pharmaceutical, Pola Pharma, Nihon Pharmaceutical, Smith & Nephew, and Sato Pharmaceutical outside the submitted work; personal fees and nonfinancial support from Janssen Pharmaceutical outside the submitted work; grants from Japan Blood Products Organization, Mochida Healthcare, Oshimatsubaki, and Shionogi outside the submitted work; and personal fees from Chugai Pharmaceutical outside the submitted work. M. Higashiyama reports grants from Kyowa Kirin during the study period; personal fees and nonfinancial support from LEO Pharma outside the submitted work; and personal fees from Kyowa Kirin, AbbVie, Celgene, Taiho Pharmaceutical, Torii Pharmaceutical, Mitsubishi Tanabe Pharma, Eli Lilly Japan, Novartis Pharma, Maruho, and Janssen Pharmaceutical outside the submitted work. Y. Hashimoto reports grants from Kyowa Kirin during the study period and personal fees from Kyowa Kirin, Eisai, AbbVie, Eli Lilly Japan, Nippon Kayaku, Janssen Pharmaceutical, Taiho Pharmaceutical, Torii Pharmaceutical, LEO Pharma, Maruho, UCB Japan, Novartis Pharma, and Celgene outside the submitted work. H. Kitabayashi is an employee of Kyowa Kirin and owns stock in the company. S. Imafuku reports grants from Kyowa Kirin during the study period; grants and personal fees from AbbVie, Eisai, Kaken Pharmaceutical, Kyowa Kirin, Sato Pharmaceutical, Sanofi, Taiho Pharmaceutical, Mitsubishi Tanabe Pharma Corporation, Tsumura, Torii Pharmaceutical, Nippon Zoki Pharmaceutical, Novartis Pharma, Maruho, and LEO Pharma outside the submitted work; grants from Pola Pharma outside the submitted work; and personal fees from Astellas, Eli Lilly Japan, MSD, Otsuka Pharmaceutical, Ono Pharmaceutical, Sun Pharma, GSK, JIMRO, Celgene, Daiichi Sankyo, Sumitomo Dainippon Pharma, Takeda Pharmaceutical, Japan Blood Products Organization, Pfizer, Bristol Myers Squibb, Meiji Seika Pharma, Janssen Pharmaceutical, and UCB Japan outside the submitted work.

## Supporting information


Appendix S1

## Data Availability

We are unable to share the data at this time because the study began enrolling patients before January 1, 2019, and informed consent does not specify data sharing, although it does mention secondary use.
