# Supplementary material for: Work productivity in real‐life employed patients with plaque psoriasis: Results from the ProLOGUE study
Source: J Dermatol. 2022 Jul 20;49(10):970–8. doi: 10.1111/1346-8138.16517 (PMC9796840; doi:10.1111/1346-8138.16517)
Supplement: Supplementary file 1 — Appendix S1 [file JDE-49-970-s001.docx]

**Appendix S1. Supplementary material**

**TABLE S1** Change from baseline in the PASI and DLQI scores in employed patients

|  | Baseline | Week 12/48 | *p* value |
| --- | --- | --- | --- |
| Employed at Week 12 (n = 48) |  |  |  |
| PASI score | 12.7 (9.3–15.6) | 0.0 (0.0–1.4) | <0.0001 |
| DLQI score | 7.0 (3.0–10.0) | 1.0 (0.5–2.0) | <0.0001 |
| Employed at Week 48 (n = 40) |  |  |  |
| PASI score | 12.7 (8.6–15.0) | 0.0 (0.0–0.8) | <0.0001 |
| DLQI score | 7.0 (3.0–10.0) | 1.0 (0.0–2.0) | <0.0001 |

Data are presented as median (Q1–Q3). *p* value was calculated using the Wilcoxon signed‑rank test.

DLQI, Dermatology Life Quality Index; PASI, Psoriasis Area and Severity Index; Q, quartile.

**FIGURE S1** Change in income opportunity loss (loss of annual income per person) owing to WPL from baseline to Week 48 in employed patients stratified by age and sex.





The top whisker represents Q3 + (1.5 × IQR) or the maximum, whichever is lower; the bottom whisker represents Q1 – (1.5 × IQR) or the minimum, whichever is higher; the top border of the box represents Q3; the bottom border of the box represents Q1; middle (bolded) line of the box represents the median.

IQR, interquartile range; Q, quartile; WPL, work productivity loss.
